# Street Collections

**Published:** 1894-09-01

**Authors:** Henry C. Burdett


### Street Collections.

By Henry C. Burdett.

[*Reprinted from the "Times" of August 25th*, 1894.]

The time has arrived when strong protest should be made against the evils and dangers of street collections in London. The hospitals of London have suffered considerably by the street collections made on one day in the year throughout London, in connection with the Hospital Saturday Fund. They have suffered because these collections irritate very many people who give largely to hospitals, and so probably lead to the withholding of much larger sums than the total amount received through their agency. They are further objectionable because they entail duties upon a large number of devoted ladies who unconsciously injure themselves by touting in this way for any cause, however worthy. In the provincial towns street collections on one day in the year have been tolerated as an advertisement for the Hospital Saturday movement, but the most thoughtful observers are of opinion that even as an advertisement the street collection has proved in practice to do at least as much harm as good. The working classes to whom the Hospital Saturday Fund particularly appeals, are wisely jealous of any outside interference, and the most worthy members of that class realise that there is a loss of dignity about street collections which irritates them and so causes many workshops to look with doubt, and sometimes even with disgust, upon the street collections of the Saturday Fund, because these methods lay it open to the criticism that the contributions thus received are not, after all, working men's gifts, pure and simple.

I should not, however, have troubled you with this letter were it not for the circumstance that individual charities are now pushing these street collections in a way which is calculated to promote dishonesty and to injure the voluntary system of support throughout the length and breadth of the land. The institution of a Lifeboat Saturday in one district of the country to meet a special emergency was little open to criticism, but the extension of a Lifeboat Saturday throughout the country would be an unmixed evil, and tend, I believe, to diminish the income of the Royal National Lifeboat Institution to a greater extent than its managers have any idea of. When, however, an occasion like the Oxford and Cambridge cricket match at Lord's is utilized for begging purposes by a so-called hospital, which I purposely do not name, and which is so inefficiently administered as to render it necessary for the Distribution Committee of the Metropolitan Hospital Sunday Fund to refuse it a grant, the time has surely come to warn the public against giving anything to these street collectors who thrust boxes into the faces of the passers-by, labelled with the name of a particular institution. At Lord's there were very great complaints of the nuisance caused by the persistent begging of the representatives of this so-called hospital, and, unfortunately, the annoyance led a number of people to denounce the whole of the hospitals of London, because they did not realise that the proceedings they justly complained of were altogether apart from the control of the managers of the reputable hospitals, who are as opposed to this system of street collections as I am myself.

To-day, Saturday, the 11th August, I notice in your columns an advertisement to the effect that a street collection for waif children will be made throughout London. London appears, therefore, to be threatened with a street collection not only upon every Saturday, but, possibly, upon every day in the year, and these collections will not be organised by representative committees of citizens, nor necessarily even by the committees of particular institutions, but they may be, as the proceedings in the police-courts have proved over and over again, but an attempt upon the part of some needy beggar to raise the wind by masquerading in the clothes of a reputable institution.

Can nothing be done to stop so gigantic an evil as these street collections threaten to become, seeing that they have already tended to lessen the contributions to reputable hospitals and institutions by irritating those who give to charities, and that the time cannot be far distant when the springs of charity may be temporarily, if not entirely, closed by these meretricious methods of raising the wind? In some of the large towns bye-laws have been made with regard to the proceedings of those who frequent the streets, and I hope it may be possible for the London County Council, and for other local authorities, to make bye-laws for London which shall regulate the nuisance of street collections. If the County Council and other authorities do not at present possess sufficient powers to free the public from the nuisance in question, I hope they may speedily be obtained. If such powers are sought, it would be a public service if the County Council would further proceed in the direction of securing the authority of Parliament to prevent any one from setting up what they may please to call a "hospital" anywhere in the metropolitan area, whether the institution be wanted or not. Such ventures are generally an attempt upon the part of an individual to promote his own ends at the expense of the citizens.

But, apart from any action by the authorities, I would urge the public to take the matter up on their own behalf. Let every one who is badgered in the streets by collectors of high or low degree not only refuse to contribute anything to the boxes, but, at the same time, make a note of the charity in whose name the nuisance is perpetrated, and then send their contributions elsewhere for the future. In this way street collections and all the evils attaching to them would speedily disappear, because they would cease to pay.

